# Emergencies

**Published:** 1871-12

**Authors:** 


### EMERGENCIES.

To know what to do in the great emergencies to which all of us are liable, the mind ought to be fully impressed, in its ordinary state, with the idea of the course to be pursued, so that the best thing to be done will be instinctively carried out, almost before the thought comes to us. When we trip the foot and fall forward, flat on the ground, the hands fly upwards first, are then spread out, and the arms stretched forward, so that the palms shall present the broadest surface to the earth, and thus best protect the face; this is instinct. The best thing to be done in an emergency will be done, if while in our calmer moods we inform ourselves of it, and have it definitely, intelligently, and understandingly impressed on the mind.

### SUFFOCATING SMOKE

Is generated in a burning building. When you are waked up at midnight by the cry of "Fire!" instantly throw yourself flat on the floor, your face almost touching it, and crawl towards the door or window, because the heated smoke is lighter than the air, and rises rapidly towards the ceiling.

A wet handkerchief, or better, woolen fabric, thrown over the head so as to fall over the face, gives admirable relief in thick smoke; but few persons nowadays carry wetted handkerchiefs about them, and while trying to hunt one up, the man might be smothered to death.

### BURSTING A BOILER

Drives out the scalding steam, which, if breathed once, so inflames the lungs as to produce certainly fatal effects. In this case, also, throw yourself on the floor, or wrap the head and face in some woolen fabric, carpet or blanket, or any other woolen material as the next best substitute. At all events keep the mouth resolutely closed, and if the steam is breathed through the nostrils, they can bear it better than the throat; as by going through the circuit of the head, the scalding air is tempered before it gets to the throat and lungs.

### SCALDS AND BURNS.

Plunge the parts into water instantaneously and keep them under, until other relief can be had; the water or any other fluid — milk, or sweet oil, or glycerine — gives relief by wholly excluding the air. It is the oxygen of the air which causes the pain.

### FIRE! RUN!

If a man asks you to go his security, cry "Fire!" and run for your life. If, however, you have the

### RHEUMATICS

Or are so fat you can't run, fall flat on the ground, shut your eyes, grind your teeth, get up a foam in the mouth, and, thinking you have hydrophobia, he will make tracks forty feet apart, as if a mad dog were after him. Better do that than have your property sold by the sheriff, and your family turned houseless and homeless into the street.

### DROWNING.

A person who cannot swim may "tread water;" keep the feet moving up and down with sufficient rapidity to keep the head above the surface, the body being perpendicular.

Another plan is to throw one's self on the back, clasp the hands under and downwards, and throw the head far enough back to leave only the nose projecting, the mouth shut; a person may thus float on the water for an hour or more. It would be well for all sea-bathers to practice these manœuvres; by making themselves adepts life may be saved. Good swimmers often rest themselves by floating or treading the water.

### SNEEZING

Is delightful, but in many cases it is very much out of place; the most certain and available method of forestalling a sneeze, without attracting attention, is to press the back of the half-bent forefinger between the upper lip and front teeth, up against the partition of the nostrils. It will never fail, if not put off until the sneeze has got a start.

### POISON.

Something needs to be done in almost all cases before a doctor can be had; if a scalding sensation is experienced in the throat, it is most likely an acid poison, and disorganizes or destroys the covering or lining or flesh of the parts, setting up destructive inflammation on the instant, and very speedily fatal from its violent action on the stomach; this is especially the case with metallic poisons; swallow instantly half a pint of sweet oil — that is best; if not at hand, melted lard or butter, the object being to dilute the poison and spread the oil as a protective coating over the inner lining of throat, stomach, and bowels.

### LAUDANUM

or other anodyne is sometimes taken by mistake or otherwise, in excess. Swallow strong coffee or the whites of several eggs instantly; all these are things to be done while the doctor is coming. Let every family remember that sweet oil, the whites of eggs, and strong coffee, antagonize a larger number of poisons than perhaps all other things together.

If laudanum or any other poison, not burning the throat, is taken and is promptly discovered, the best plan is to get it out of the stomach instantly, which is done by stirring a tablespoon of ground mustard in a tumbler of water, and drinking it down at once; almost before it is down the whole contents of the stomach begin to be ejected.

